# Supplementary material for: Evaluating Diagnostic Ultrasound of the Vagus Nerve as a Surrogate Marker for Autonomic Neuropathy in Diabetic Patients
Source: Medicina (Kaunas). 2023 Mar 8;59(3):525. doi: 10.3390/medicina59030525 (PMC10058247; doi:10.3390/medicina59030525)
Supplement: Supplementary file 1 [file medicina-59-00525-s001.zip › medicina-2236486-supplementary.pdf]

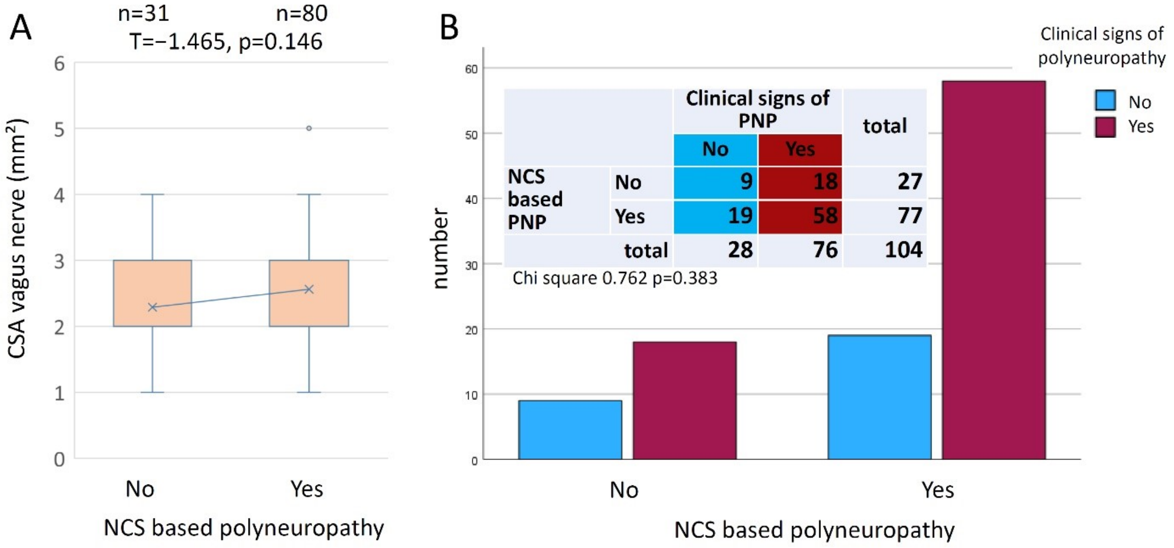

Supplementary Figure S1. Nerve conduction studies (NCS). Diagnosis of polyneuropathy was done if at least two nerves showed pathological measurements. A) Cross-sectional area (CSA) of the vagus nerve did not differ significantly between diabetes patients with and without NCS based diagnosis of polyneuropathy. B) Comparison between clinically defined diagnosis and NCS based diagnosis of polyneuropathy. 19 patients did not have clinical signs of polyneuropathy but met NCS based diagnosis criteria for polyneuropathy, which may represent subclincal neuropathy. 18 patients had clinical signs but did not meet NCS based diagnosis criteria, which may possibly be due to small fiber neuropathy.

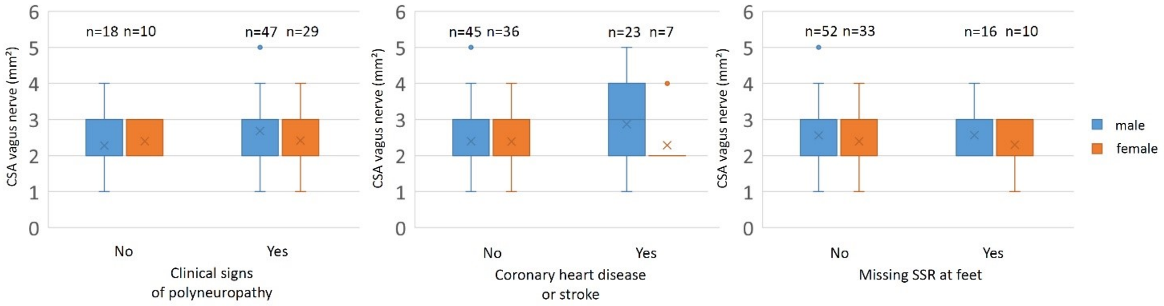

Supplementary Figure S2. Box plots of vagus nerve CSA in different subgroups of patients with diabetes separated according to gender. Statistical tests were not done due to relatively small patient numbers in some of the subgroups.

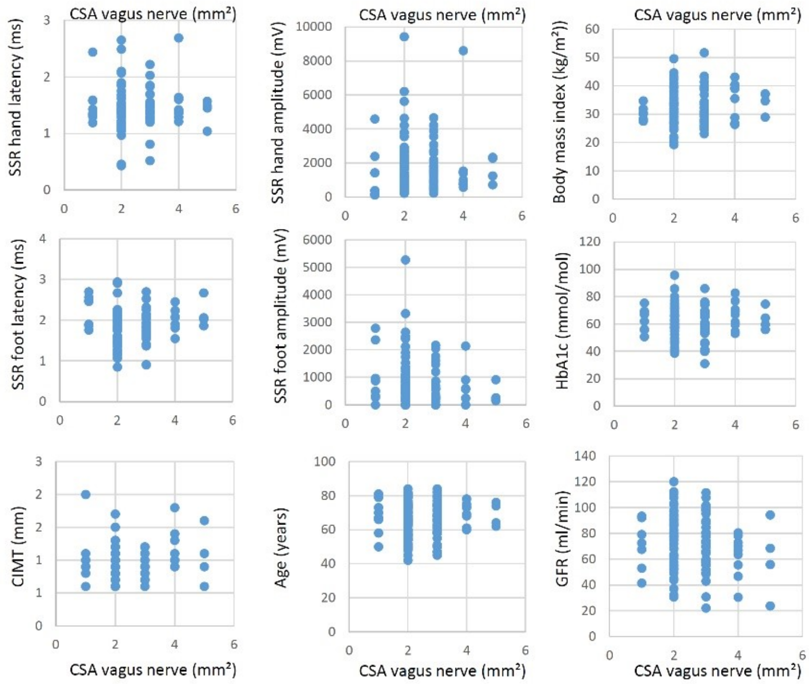

Supplementary Figure S3. Scatterplots of vagus nerve cross-sectional area (CSA) and all of the parameters analyzed (sympathetic skin response (SSR), carotid intima-media thickness (CIMT), age, body mass index (BMI), HBA1c, and glomerular filtration rate (GFR). None of these parameters showed any statistically significant correlation.

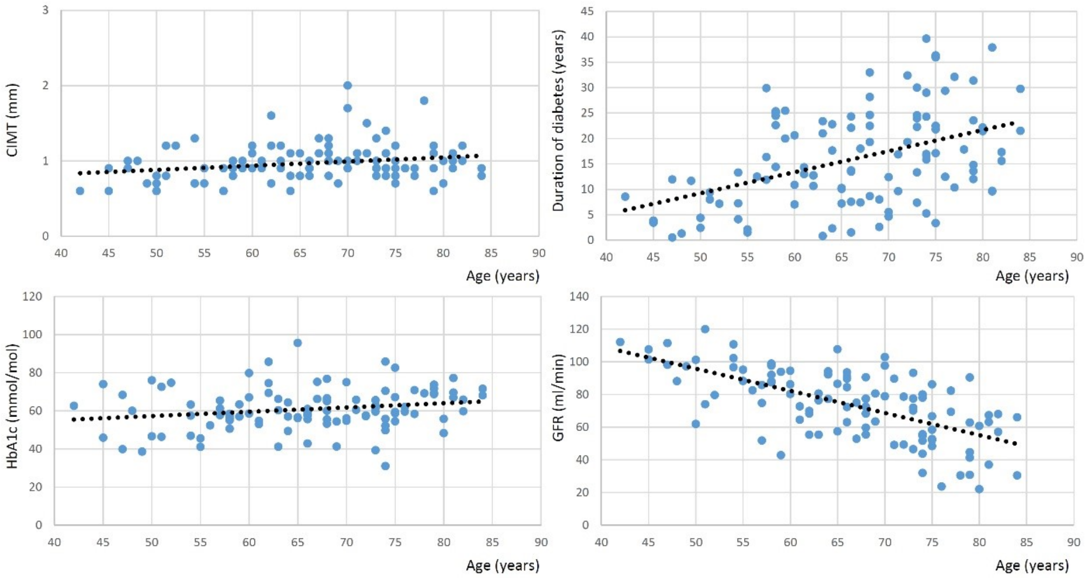

Supplementary Figure S4. Scatterplots of age and carotid intima-media thickness (CIMT), duration of disease, HBA1c, and glomerular filtration rate (GFR), which revealed statistically significant correlations.

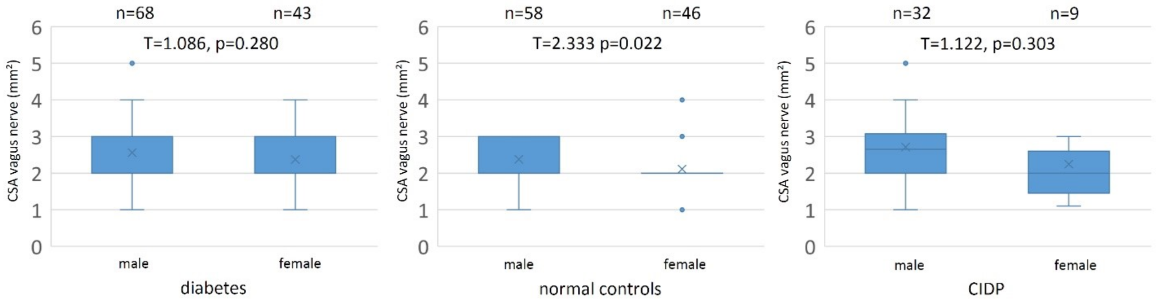

Supplementary Figure S5. Box plots of vagus nerve cross-sectional area (CSA) in patients with type 2 diabetes, normal controls, and patients with chronic inflammatory demyelinating polyneuropathy (CIDP) separated according to gender. Vagus nerve CSA showed a statistically significant sex dependent difference for the normal controls (p=0.022).

Supplementary Table S1. Regression analysis using cross-sectional area (CSA) of the vagus nerve as dependent variable and sympathetic skin response (SSR) parameters and carotid intima-media thickness (CIMT) as independent variables.

	**Regression coefficient B**	**Standard error**	**Beta**	**T**	**p**
constant	2.613	0.732		3.432	<0.001
SSR hand latency	-0.045	0.291	-0.018	-0.154	0.878
SSR hand amplitude	0.000008412	0.291	0.015	0.129	0.897
SSR foot latency	0.000	0.000	-0.198	-1.698	0.093
CIMT	0.075	0.428	0.020	0.174	0.862

Adjusted $R^2 = -0.018$

Squaresum 3.180, $F = 0.710$, $p = 0.617$
